# Titania nanospikes activate macrophage phagocytosis by ligand-independent contact stimulation

**DOI:** 10.1038/s41598-022-16214-2

**Published:** 2022-07-18

**Authors:** Nadia Kartikasari, Masahiro Yamada, Jun Watanabe, Watcharaphol Tiskratok, Xindie He, Hiroshi Egusa

**Affiliations:** 1 Division of Molecular and Regenerative Prosthodontics, Tohoku University Graduate School of Dentistry, 4-1 Seiryo-machi, Aoba-ku, Sendai, Miyagi 980-8575 Japan; 2 Center for Advanced Stem Cell and Regenerative Research, Tohoku University Graduate School of Dentistry, Sendai, Miyagi Japan

**Keywords:** Nanoscale materials, Phagocytes

## Abstract

Macrophage phagocytosis is an important research target to combat various inflammatory or autoimmune diseases; however, the phenomenon has never been controlled by artificial means. Titania nanospikes created by alkaline etching treatment can tune macrophage polarization toward a M1-like type and might regulate macrophage phagocytosis. This in vitro study aimed to determine whether the two-dimensional titania nanosurfaces created by alkaline etching treatment activated the macrophage phagocytosis by nanospike-mediated contact stimulation. On two-dimensional pure titanium sheets, alkaline etching treatments with different protocols created superhydrophilic nanosurfaces with hydroxyl function groups and moderate or dense nanospikes. Both types of titania nanosurfaces promoted the phagocytic activity of the mouse macrophage-like cell line, J774A.1, through upregulation of M1 polarization markers and phagocytosis-related receptors, such as toll-like receptors (TLR2 and 4). In contrast, the hydrophobic smooth or micro-roughened titanium surfaces did not activate macrophage phagocytosis or the expression of related receptors. These phenomena remained unchanged even under the antibody blockade of macrophage TLR2 but were either suppressed or augmented for each surface excited by ultraviolet irradiation. Titania nanospikes induced paxillin expression and provided physical stimuli to macrophages, the extent of which was positively correlated with TLR expression levels. Ligand stimulation with lipopolysaccharide did not upregulate macrophage TLR expression but further enhanced M1 marker expression by titania nanosurfaces. These results showed that the two-dimensional titania nanosurfaces activated macrophage phagocytosis by enhancing expression of phagocytosis-related receptors through nanospike-mediated contact stimulation, in assistance with physical surface properties, in a ligand-independent manner.

## Introduction

Phagocytosis is a crucial innate immunity mechanism that eliminates pathogens from an infected site. Phagocytosis slows the diffusion of pathogens and reduces subsequent inflammatory reactions in local tissues^1^. Professional phagocytes such as neutrophils, monocytes, and monocyte-derived or tissue-resident macrophages play a central role in phagocytosis^2^. Macrophage phagocytosis cleans not only microbes, but also dying neutrophils and fights pathogens as the first response^1^. This phenomenon is involved in the initiation and resolution of inflammation in the early phase, the failure of which leads to prolonged and eventually chronic inflammation. Macrophages can accumulate around biomaterials and control local inflammatory responses and wound healing^3^. Furthermore, macrophage phagocytosis is strongly associated with the inhibition of tumor invasion and plays an important role in the induction of antitumor immunity by bridging innate and adaptive immunity^4^. Therefore, the technology to tune macrophage phagocytosis has attracted attention for the development of therapeutic and diagnostic tools for a large variety of biological events, such as inflammatory and oncological diseases, or tissue regeneration^5,6^.

Toll-like receptors (TLR2 and 4) are localized to the cell surface and respond to pathogen-associated molecular patterns (PAMPs) for microbial recognition^7^. TLR2 mainly recognizes lipoproteins, peptidoglycans, lipoteichoic acid, and zymosan (fungal glucans), while TLR4 recognizes lipopolysaccharides (LPS) ^8^. TLR activation in macrophages promotes phagocytosis for microbial clearance and antigen presentation ^9^. TLR2 and 4 can also recognize titanium particles containing PAMPs^10^, the prolonged exposure to which causes wear debris-mediated peri-implant osteolysis^11^. In addition, scavenger receptors (SRs), such as scavenger receptor-A (SR-A or CD204) or macrophage receptor with collagenous structure (MARCO), are involved in the recognition of diverse types of ligands that include biological organic components, as well as unopsonized artificial particles^12,13^. TLRs interact with SRs and enhance phagocytosis^9^. Controlling the expression of these receptors is a key to the activation of macrophage phagocytosis.

The functions of immune cells are regulated not only by biochemical molecules, but also by physical stimulation from the surrounding microenvironments^14^. Focal adhesion plaques sense the microenvironment and regulate macrophage polarization and function via mechanotransduction systems that involve focal adhesion kinases and actin contraction^15^. Compared to ligand stimulation by biochemical molecules, mechanotransduction systems activated by physical stimulation may have a greater impact on the regulation of macrophage function^16^. Mechanotransduction systems are also involved in TLR signaling pathways in macrophages^17^. The surface properties of biomaterials can physically stimulate the cells in microenvironments to regulate their functions^18^. Two-dimensional (2D) patterned platforms consisting of one-dimensional (1D) vertical nanostructures have recently attracted attention for their immunomodulatory effects^19^. Dimethylpolysiloxane micropillars, for instance, activate cytotoxic T lymphocytes, as shown by the increased expression of interferon-gamma (IFN-γ)^20^ or by the enhanced killing ability of target cells^21^. Zinc oxide-based nanowires augmented the major class I histocompatibility complex-induced expression of lysosomal-associated membrane protein-1 in natural killer cells^22^. These types of 2D patterned platforms with vertical 1D nanostructures deform the cell wall or cell membrane, inducing various biological events inside cells or microorganisms^23,24^. However, no 2D patterned platforms have been used as an artificial environment to regulate the phagocytic activity of professional phagocytes.

Alkali etching treatment with concentrated sodium hydroxide creates numerous and dense titania (TiO_2_)-based nanospikes on titanium substrates^25,26^. Such titania nanospikes may physically stimulate macrophages and tune them to resist infection. Titania nanospiked particles promote the macrophage inflammatory response through physical activation and function as an adjuvant for immunomodulation, in conjunction with TLR4 agonists^5^. Titania nanospikes created on 2D titanium substrates by alkali etching treatment polarize macrophages into an M1-like type and tune them to produce inhibitory factors for osteoclast differentiation^27^. Even in other types of cells, titania nanosurfaces with nanospikes changed cell morphologies and augmented their inherent functions without any adverse events^25,26^. These observations suggest that titania nanosurfaces created by alkaline etching treatment regulate phagocytic activities of macrophages through a mechanotransduction system by nanospike-induced contact stimulation. This in vitro study aimed to determine whether the 2D titania nanosurfaces created by alkali etching treatment regulates phagocytic activities and the expression of related receptor expressions in macrophages by nanospike-mediated contact stimulation.

## Material and methods

### Titanium sample preparation

Commercially pure grade I 0.18 mm-thick titanium sheets (Nishimura Co., Ltd., Fukui, Japan) with a smooth (SM) surface were used as the culture substrate. They were cleaned with acetone, ethanol, and ultrapure water under ultrasonication. An SM titanium sheet with an acid-etched surface, as a representative clinically used micro-roughened (MR) surface, was prepared by immersing the sheet in 67% (w/w) sulfuric acid solution (FUJIFILM Wako Pure Chemical Corporation, Osaka, Japan) at 120 °C for 75 s^25^. Two types of nano-roughened (NR) surfaces were prepared using alkaline etching treatment, as described previously^25,26,28^. Briefly, SM titanium sheets were boiled in sodium hydroxide for 24 h, washed with ultrapure water, air-dried overnight, sintered in a furnace at 600 °C for 1 h with a steadily increasing temperature (5 °C/min), and finally air-cooled. A 5 M sodium hydroxide solution at 60 °C was used for the NR1 surface and a 10 M solution at 90 °C for the NR2 surface. Porous and spiky titania nanolayers with a thickness of several hundred nanometers are formed on titanium sheets after the alkaline etching treatment^27^.

To determine the surface properties that affect macrophage responses, titanium samples with hydrophilic treatment with no changes made to the surface topography were prepared as controls according to a previously reported method^27^. To excite the superficial oxide layer on the titanium or titania surface through the photocatalytic effect^29^, ultraviolet (UV) rays were irradiated onto the SM, NR1, and NR2 surfaces for 3 days, using a TFL-40 V UV transilluminator (UVP, Cambridge, UK) with a peak wavelength of 354 nm (UV-A region) in high-intensity mode. Except for the samples subjected to UV treatment, all other samples were autoclaved immediately before use. UV-irradiated samples were subjected to surface analyses or cell seeding immediately after the treatment.

### Analyses of surface properties

The surface topography of the titanium sheets was evaluated using an XL30 scanning electron microscope (SEM; Philips, Eindhoven, Netherlands). Vertex extraction images of the MR, NR1, and NR2 surfaces were obtained from 30,000 × magnification SEM images, by extracting the bright spots using a WinRoof image analyzer (MITANI Corporation, Tokyo, Japan). Spike density was calculated by counting vertex spots per unit micrometer area.

Heights of the nanospikes were evaluated using vertical roughness parameters: the arithmetical mean height (Sa) and maximum peak height (Sp). These parameters were measured at a length of 120 μm on each surface using a Talysurf PGI 1250A laser microscope (Taylor Hobson, Ametek, Leicester, UK) with waviness removal by approximation of a cubic polynomial.

The surface energy of each titanium sheet was determined by measuring the water contact angle of 30 μL of distilled water (DW) using a CA-X sessile drop machine (Kyowa Interface Science Co. Ltd., Saitama, Japan)^27^. A water contact angle > 90° was defined as hydrophobic; < 90°, hydrophilic; and < 10°, superhydrophilic^30^.

Fourier transform infrared (FTIR) spectra of the surfaces were obtained using an IRT7000 linear array imaging microscope (JASCO Corporation, Tokyo, Japan). The microreflection spectrum was recorded in the range of 4000–2000 cm^−1^ at a spectral resolution of 4 cm^–1^, 500 accumulations, and 50 μm^2^ aperture. Background correction was performed based on the surface spectrum of SM.

### J774A.1 cell culture

The mouse macrophage-like cell lines (JCRB9108, J774A.1) obtained from the Japanese Collection of Research Bioresources Cell Bank (National Institutes of Biomedical Innovation, Health and Nutrition, Tokyo, Japan) were cultured in Dulbecco’s modified Eagle’s medium (DMEM; Nacalai Tesque, Kyoto, Japan) supplemented with 10% fetal bovine serum (FBS; Japan Bioserum, Hiroshima, Japan), 200 mmol/L L-glutamine solution (FUJIFILM Wako Pure Chemical Corporation), 100 U of penicillin, and 100 μg/mL of streptomycin solution (FUJIFILM Wako Pure Chemical Corporation) at 37 °C in humidified 5% CO_2_^27^. After 80% confluency, the cells were detached via scraping without any detachment reagents. They were seeded at a density from 2.0 × 10^4^ to 3.0 × 10^5^ cells/mL in 6-, 12-, or 96-well polystyrene culture plates with or without titanium sheets in DMEM with the same supplements as previously mentioned. The cells were also cultured in DMEM without FBS to evaluate cellular responses on each substrate under serum-free conditions. The cells were cultured for 1 or 3 days.

According to our previous protocols^27^, an M1 control of J774A.1 cells was induced using 100 ng/mL of LPS from *Escherichia coli* O55:B5 purified by phenol extraction (Sigma-Aldrich, St. Louis, MO, USA), and 10 ng/mL of mouse recombinant IFN-γ (FUJIFILM Wako Pure Chemical Corporation) for 24 h, while an M2 control was induced using 20 ng/mL of mouse recombinant IL-4 (FUJIFILM Wako Pure Chemical Corporation) for 24 h.

### Stimulation and blocking of TLRs in macrophages

To analyze the effects of TLR4-ligand stimulation with time on TLR2 and TLR4 expression in macrophages, J774A.1 cells were seeded at a density of 2.0 × 10^5^ cells/mL on a 6-well polystyrene culture plate and incubated in DMEM with or without the M1-inducing reagents for 3, 6, 12 and 24 h, as described previously.

To analyze the involvement of TLR2-ligand stimulation in the activation of macrophages by titanium or titania surfaces, J774A.1 cells were seeded at a density of 2.0 × 10^5^ cells/mL on a 6-well culture plate with or without titanium sheets and incubated in DMEM with or without 10 µg/mL of anti-TLR2 recombinant antibody (Cat. #153,002, Biolegend, San Diego, CA, USA) for 1 h. Following 1 h of incubation, the culture was further incubated in fresh DMEM, with or without M1-inducing reagents, for 24 h. The validity of the anti-TLR2 antibody concentration and co-culture time was assessed in an independent J774A.1 culture using the TLR2 ligand zymosan (Cat. #: NBP2-26,233, Novus Biological, Oxford Abingdon, UK). The appropriate concentration of zymosan and the co-culture time were determined for the validation of blocking conditions with the anti-TLR2 antibody. J774A.1 cells were seeded at a density of 2.0 × 10^5^ cells/mL on a 6-well polystyrene culture plate and incubated in DMEM with 0, 1, 5, 10, and 20 µg/mL zymosan for 12 and 24 h.

To analyze the synergistic effects of ligand stimulation and titanium or titania surfaces on macrophages, J774A.1 cells were seeded at a density of 2.0 × 10^5^ cells/mL on a 6-well culture plate with or without titanium sheets and incubated in DMEM with or without LPS for 24 h. The LPS concentration required for ligand stimulation was determined in an independent J774A.1 culture in a 6-well polystyrene culture plate using 0, 100, 500, and 1,000 ng/mL.

### Reverse transcription-polymerase chain reaction (RT-PCR) analysis

Total RNA from J774A.1 cell culture was extracted using TRIzol reagent (Ambion/Life Technologies, Carlsbad, CA, USA). RNA isolation and purification were performed using an RNAeasy Mini Kit (Qiagen, Hilden, Germany), followed by DNase treatment (Thermo Fisher Scientific, Waltham, MA, USA). Complementary DNA (cDNA) was synthesized using a PrimeScript™ II 1st Strand cDNA Synthesis Kit (Takara Bio, Shiga, Japan)^27^. Messenger RNA (mRNA) expression was determined using a Thunderbird SYBR qPCR Mix (Toyobo, Osaka, Japan) for SYBR green-based RT-PCR on a StepOnePlus Real-Time PCR system (Applied Biosystems, Thermo Fisher Scientific). The target gene expression levels were quantitatively analyzed using the comparative cycle time (ΔΔCT) method. Glyceraldehyde 3-phosphate dehydrogenase (*GAPDH*) was used as a housekeeping gene. The 2^ΔΔCT^ method was used to calculate the fold-changes in gene expression in each experimental sample compared to untreated polystyrene. The primers used are listed in Table S1.

### Immunofluorescent staining

On day 1, J774A.1 cells cultured on polystyrene, titanium, or titania surfaces were fixed with 10% neutral buffered formalin solution. After washing with phosphate-buffered saline (PBS), non-specific binding proteins were blocked using blocking buffer, containing 3.0% BSA, 0.1% Tween 20, and 0.1% Triton-X, for 60 min. The cells were incubated with rabbit anti-inducible nitric oxide synthase (iNOS) polyclonal primary antibody (ab3523, Abcam, Cambridge, UK), mouse anti-TLR4 monoclonal (SC-293072, Santa Cruz Biotechnology, Inc., Dallas, TX, USA), or rabbit anti-paxillin monoclonal (ab32084) primary antibodies at 1/200, 1/100, or 1/250 dilution, respectively. The cells were incubated in the dark at 4 °C overnight, washed with PBS, and incubated again for 1.5 h in the dark, in a mixture solution consisting of 1/200 diluted Alexa Fluor 488 goat anti-mouse (A11001, Thermo Fisher Scientific) or anti-rabbit (ab150081, Abcam) secondary antibody, 1/400 diluted rhodamine-phalloidin for F-actin staining, and 1/500 diluted Hoechst 33,258 for nuclear staining. After washing with PBS, cells were mounted with 90% glycerol and observed under an LSM 780 confocal laser microscope.

### WST-1 and endotoxin assays

To evaluate the cytotoxicity of each prepared surface, the number of adherent cells was quantified using the tetrazolium salt WST-1 reagent (Cell Proliferation Reagent WST-1, Roche Diagnostics Deutschland GmbH, Mannheim, Germany). which forms colored formazan products by reacting with nicotinamide adenine dinucleotide phosphate found in viable cells. Cell grown for 24 h were incubated with a fresh culture medium containing the WST-1 reagent for 4 h at 37 ℃ in a 5% CO_2_ atmosphere, and the absorbance was read at 450 nm using an ELISA reader.

To quantify the endotoxins present on each surface, titanium samples without cells were immersed in a limulus amebocyte lysate reagent (Limulus Color KY Test Wako, FUJIFILM Wako Pure Chemical Corporation) containing recombinant factor C and an inhibitor of β-d-glucan-sensitive factor G. This analysis is based on the sequential reactions in which endotoxins activate the serine protease precursor and the coagulation enzyme, finally hydrolyzing the yellow color-developing synthetic substrate (Boc-Thr-Gly-Arg-pNA) and releases a color-developing group (pNA). The samples were reacted with the reagent for 15 min, and the absorbance was read at 405 nm using an ELISA reader. The endotoxin concentration on each surface was determined based on a calibration curve using an endotoxin standard solution.

### Phagocytosis assay

Macrophage phagocytic activity on polystyrene and titanium or titania surfaces was evaluated using fluorescent-labeled 1 μm-diameter microbeads (Fluoresbrite YG Microspheres 1.0, Polysciences Inc., Warrington, PA, USA) (excitation maximum: 441 nm, emission maximum: 486 nm). J774A.1 cells cultured on polystyrene and titanium or titania surfaces for 1 day were co-incubated with microbeads added to the culture media at a density of 20 particles/cell for an additional 4 h. The cells were washed twice with PBS and fixed with 10% neutral buffered formalin solution for 10 min. The cells were washed three times with PBS and stained with rhodamine-phalloidin, as described in the previous section. A cover glass was placed on the culture on polystyrene and titanium or titania surfaces with a mounting medium containing 4′,6-diamidino-2-phenylindole (DAPI; VECTASHIELD H-1200, Vector Laboratories, Inc.). Cultures were observed under a BZ-X810 fluorescence microscope (Keyence corporation, Osaka, Japan). Green, light blue, or blue dot signals overlapping with the cell body in the merged images were defined as phagocytized microbeads. The number of microbeads per unit cell was measured using a WinRoof image analyzer.

### Cell morphometry on titanium surfaces

On day 1, J774A.1 cells cultured on titanium or titania surfaces were fixed in 10% neutral buffered formalin for 30 min. The samples were washed with DW, air-dried, and then sputter-coated with a gold/palladium alloy. SEM analyses were performed on the cells using an XL30 system (Philips, Eindhoven, Netherlands) at an acceleration voltage of 10 keV. Linear or point parts with the same high brightness as the vertices of the titanium or titania surfaces in 10,000 × magnification SEM images of the peripheral region of the cell body were detected using the same method as above. The shining spots were defined as the spots where the vertices of titanium or titania spikes dug into the cell body until the vertex shapes were visible. The area ratio of the shining spots to the visual field was measured using an image analyzer.

#### Statistical analysis

For surface analysis on titanium sheets, three independent samples on each titanium or titania surface were subjected to a series of measurements. Six independent samples were subjected to the endotoxin assay. All culture experiments, except for phagocytosis and immunofluorescence assays, were performed on at least three independent cell batches (*N* = 3 or 4). Gene expression analyses were performed thrice, and representative datasets were presented after confirming consistency (*N* = 3). Immunofluorescence assays for TLR4 and paxillin expression were performed on 12–14 cells (*N* = 12–14) and 15–52 cells (*N* = 15–52), respectively, at multiple spots randomly selected from a single cell culture. The number of beads per unit cell was also measured at multiple spots randomly selected from a single cell culture, where cells (*N* = 68–358) were used regardless of bead uptake. Assays with a single cell culture were repeated on different days, to confirm consistency of results. One-way analysis of variance (ANOVA) was used to assess differences among multiple experimental groups, while two-way ANOVA was used to assess the interactions between differences in the substrate types and culturing periods, applied reagents, or wettability adjustments. When appropriate, post-hoc Tukey’s honestly significant difference (HSD) test, Bonferroni’s multiple comparison test, the Games–Howell test, or Dunnett’s test were used. Statistical significance was set at* P* < 0.05. All statistical analyses were performed using the IBM SPSS Statistics version 21 (IBM Japan, Ltd., Tokyo, Japan)^27^.

## Results

### Topographical and physicochemical features of titania nanosurfaces

**Figure 1:**
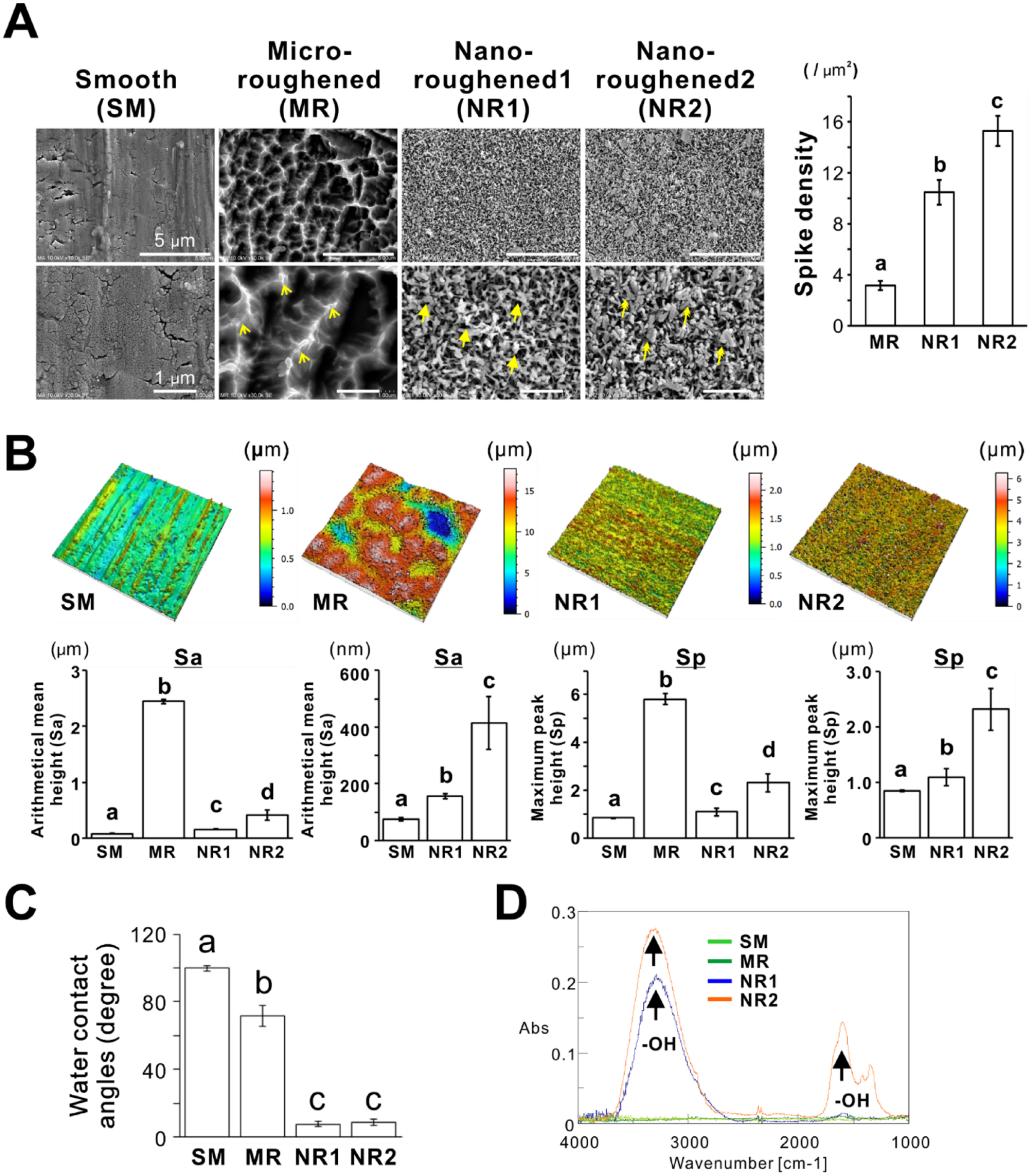
Topographical and physicochemical features of titania nanosurfaces (**A**) Representative scanning electron microscope (SEM) images of titanium sheet with smooth (SM), micro-roughened (MR), and nano-roughened (NR) 1 and 2 surfaces. The histogram indicates spike density evaluated by counting the vertices per unit micron area on the MR, NR1, and NR2 surface. (**B**) Representative birds-eye views (upper panels) and vertical roughness parameters (lower histograms) such as Sa and Sp, determined by a laser microscope, (**C**) water contact angles determined by a sessile drop method, and (**D**) FTIR spectra of each type of surfaces. Yellow arrowheads, arrows, and double arrows in (**A**) high magnification SEM images indicate sharp ridges on the MR titanium surface and multiple nanospikes on NR1 and NR2 titania surfaces, respectively. Black arrows in (**D**) FTIR spectra indicate the peak of hydroxyl groups (-OH). Data presented as mean ± standard deviation (SD; *N* = 3). Different letters indicate statistically significant differences (*P* < 0.05; Tukey’s honestly significant difference [HSD] test). Sa, arithmetical mean height; Sp, maximum pit height; FTIR, Fourier transform infrared. Note that the NR1 and NR2 surfaces showed dense nanospikes (A and B), superhydrophilicity (C), and the presence of hydroxyl groups, consistent with a previous report ^27^.

The SEM images demonstrated a relatively flat appearance with shallow microgrooves and mountain chain-like irregularities with relatively sharp ridges (Fig. 1A, arrowheads) on the SM and MR titanium surfaces, respectively. In contrast, the NR1 and NR2 titania surfaces had multiple nanospikes and a sponge-like inner network on the superficial and underlying layers. The nanospikes (Fig. 1A, double arrows) on the NR2 titania surfaces were denser than those on the NR1 surfaces (Fig. 1A, arrows). The spike density per unit micron area was less than 4 on the MR titanium surfaces, whereas the value was approximately 10 and 15 for the NR1 and NR2 titania surfaces, respectively (Fig. 1A, histogram; *P* < 0.05, Tukey’s HSD test). Roughness parameters showing the height of spikes, such as Sa and Sp, were the highest on the MR titanium surfaces with micron-level roughness (Fig. 1B; *P* < 0.05, Tukey’s HSD test). The heights of the spikes on the NR1 and NR2 titania surfaces were at the nanoscale. The NR1 titania surfaces were slightly higher in these roughness parameters than SM surfaces, whereas the NR2 titania surfaces showed two times or more roughness than the SM and NR1 surfaces (Fig. 1B; *P* < 0.05, Tukey’s HSD test).

The NR1 and NR2 showed superhydrophilicity, which was confirmed by water contact angles < 10° (Fig. 1C; *P* < 0.05, Tukey’s HSD test) and the presence of the hydroxyl group, a typical hydrophilic functional group (in their FTIR spectra; Fig. 1D). In contrast, the SM and MR titanium surfaces showed water contact angles of approximately 100° (hydrophobic) and 70° (hydrophilic), respectively (Fig. 1C), without the presence of hydroxyl group (Fig. 1D). These results were consistent with the observations in a previous study that performed the same analyses on the same types of titanium samples^27^.

### Effects of titania nanosurfaces on polarization marker expressions in macrophages

**Figure 2:**
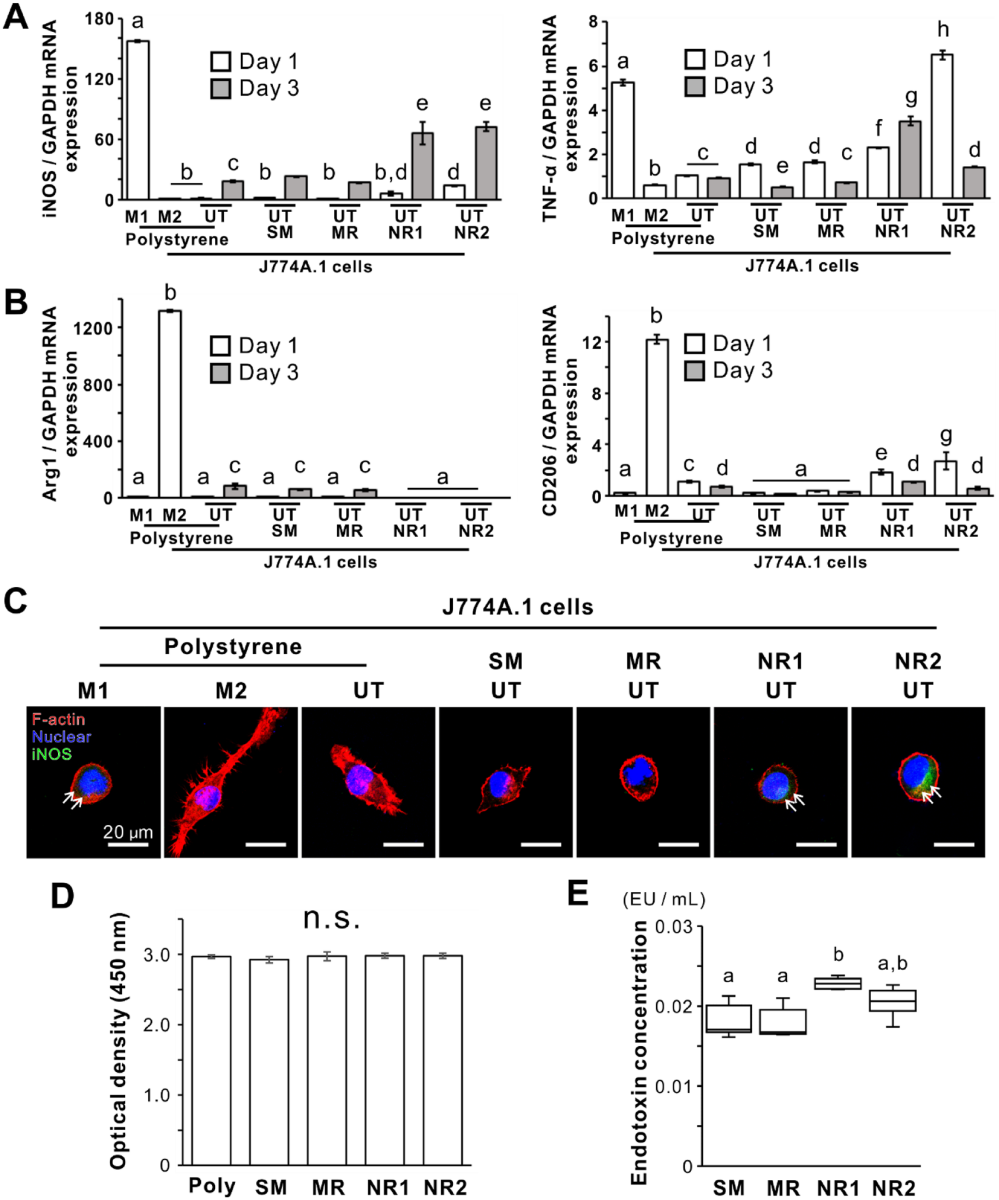
Effects of titania nanosurfaces on the expression of polarization markers of macrophages Gene expression of M1 polarization markers (**A**) inducible nitric oxide synthase (*iNOS*), tumor necrosis factor-α (*TNF-α*), and (**B**) M2 polarization markers arginase 1 (*Arg1*) and cluster of differentiation 206 (*CD206*) relative to glyceraldehyde 3-phosphate dehydrogenase (*GAPDH*) were analyzed by reverse transcription-polymerase chain reaction (RT-PCR) in J774A.1 cells cultured for days 1 and 3 on a polystyrene culture plate or smooth (SM), micro-roughened (MR), or nano-roughened (NR) 1 or 2 surfaces with or without the M1-induction. (**C**) Representative confocal laser microscopy images of F-actin (red), nuclear (blue), and iNOS (green) staining in the cells. (**D**) WST-1-based evaluation of the number of adherent cells under different culture conditions on day 1. (**E**) Concentrations of endotoxins detected on the titanium or titania surfaces without cells. Data represented as mean ± standard deviation (SD; *N* = 3 in **A, B** and **C**) or box plots (*N* = 6 in **E**). Different letters indicate statistically significant differences between them (*P* < 0.05; Bonferroni’s multiple comparison test or Tukey’s honestly significant difference [HSD] test). UT, untreated cells; M1, M1-induced cells; M2, M2-induced cells; n.s., non-significant difference.

Although not up to the levels in J774A.1 cells with M1-induction, the expression of the representative M1 gene marker *iNOS* was upregulated in the untreated cells on the NR1 or NR2 or both on days 1 and 3 compared to that in the untreated cells on polystyrene and the other titanium surfaces (Fig. 2A; *P* < 0.05, Bonferroni’s multiple comparison test). The expression of another M1 marker gene *TNF-α,* was also higher on both these titania nanosurfaces than on the other substrates consistently for three culturing days (Fig. 2A; *P* < 0.05, Bonferroni’s multiple comparison test). In particular, the *TNF-α* expression in the untreated cells on the NR2 titania nanosurface on day 1 was higher than in the M1-induced cells (Fig. 2A; *P* < 0.05, Bonferroni’s multiple comparison test). In contrast, expression of representative M2 gene markers, *Arg1* and *CD206*, was not notably upregulated in J774A.1 cells on any substrate on both days 1 and 3, compared to than in the M2-induced cells (Fig. 2B). Immunofluorescence signals for iNOS were detected in M1-induced cells and untreated cells cultured on the NR1 or NR2 surfaces on day 1 (Fig. 2C). These cells exhibited circular shapes. In contrast, iNOS signals were not detected in the M2-induced cells with elongated and spindle-like shapes and in untreated cells with polygonal or acorn-like shapes cultured on polystyrene, SM, or MR surfaces.

The number of adherent cells on day 1 did not differ between the titanium and titania surfaces (Fig. 2D; *P* > 0.05, Tukey’s HSD test). The concentration of endotoxins detected on the NR1 surface was higher than on the other surfaces (Fig. 2E; *P* < 0.05, Tukey’s HSD test) but was below 0.03 EU/mL regardless of the surface types.

### Effects of titania nanosurfaces on TLR expressions in macrophages

**Figure 3:**
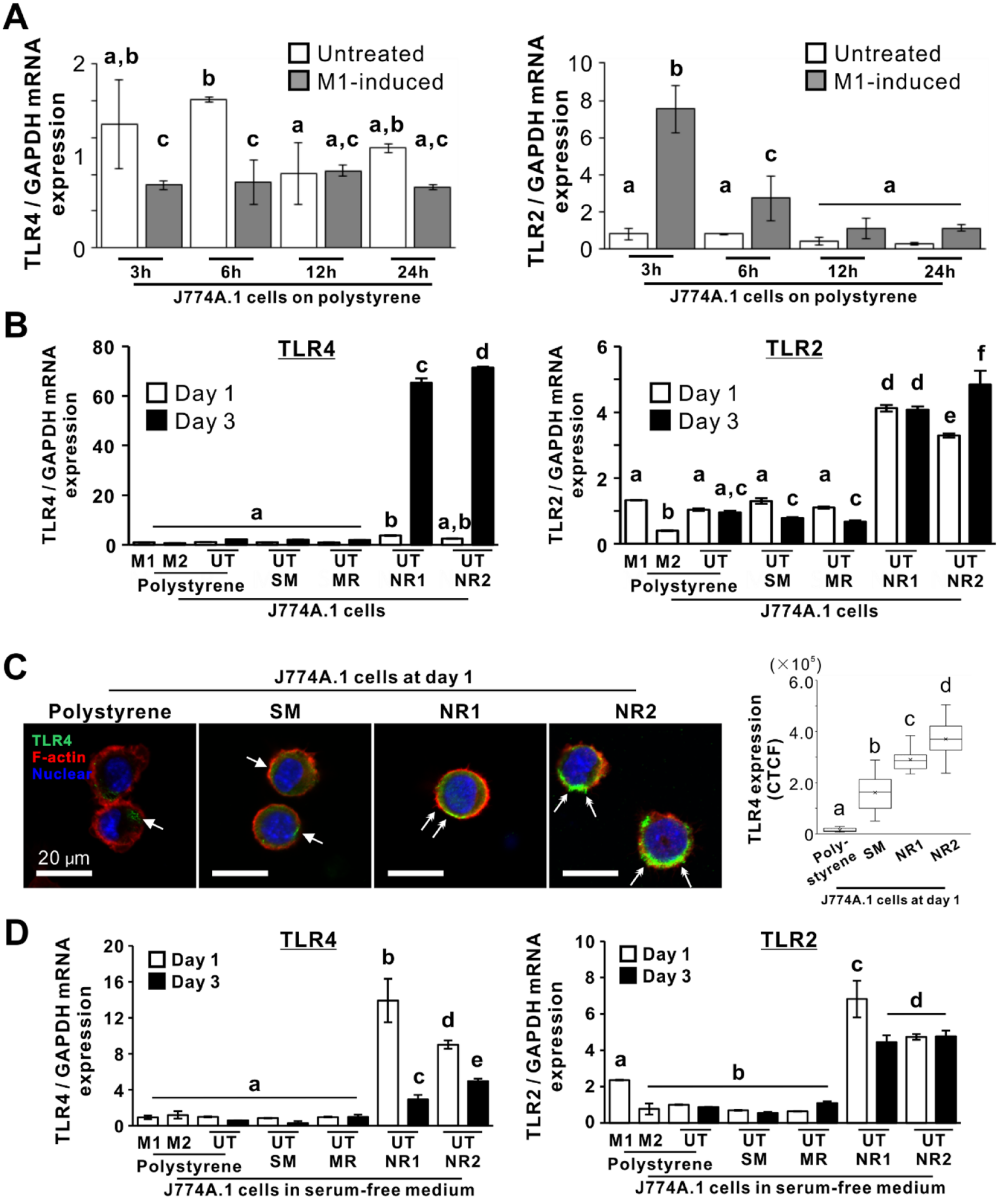
Effects of titania nanosurfaces on TLR expressions of macrophages Gene expression levels of toll-like receptor 2 (*TLR2*) and toll-like receptor 4 (*TLR4*), relative to glyceraldehyde 3-phosphate dehydrogenase (*GAPDH*), as analyzed by reverse transcription-polymerase chain reaction (RT-PCR), in J774A.1 cells cultured on a polystyrene culture plate for 3, 6, 12, and 24 h with or without M1-induction **(A)** or on polystyrene or smooth (SM), micro-roughened (MR), or nano-roughened (NR) 1 or NR2 surfaces with or without the M1-induction for days 1 and 3 **(B)**. (**C**) Representative confocal laser microscopic images of F-actin (red), nuclear (blue), and TLR4 (green) staining, and the intensity of TLR4-labeled signals in the cells under different culture conditions. (**D**) Gene expression levels of *TLR4* and *TLR2*, relative to *GAPDH* in the cells under serum-free conditions. White arrows indicate TLR4 fluorescence signals. Data represented as mean ± standard deviation (SD; *N* = 3 in **A**, **B,** and **D**) and box plots (*N* = 12–14 in **C**). Different letters indicate statistically significant differences (*P* < 0.05, Bonferroni multiple comparisons test in **A** and **B**, or Games–Howell test in **C**). UT, untreated cells; M1, M1-induced cells; M2, M2-induced cells; CTCF, Corrected total cell fluorescence. Note (**C**) the strong TLR4 signals in macrophages on the NR1 or NR2 titania surfaces (double arrows), in contrast with weaker signals on the other surfaces (arrows).

M1-induction with LPS and IFN-γ did not increase the *TLR4* expression in J774A.1 cells on a polystyrene culture plate until 24 h after seeding (Fig. 3A); however, the induction upregulated *TLR2* expression at 3 and 6 h post seeding (*P* < 0.05, Bonferroni’s multiple comparison test), and decreased after 12 h (*P* < 0.05, Bonferroni’s multiple comparison test). In contrast, both titania nanosurfaces significantly upregulated *TLR4* gene expression in untreated J774A.1 cells on day 3 (Fig. 3B; *P* < 0.05, Bonferroni’s multiple comparison test). In addition, *TLR2* gene expression on both titania nanosurfaces increased on day 1 (*P* < 0.05, Bonferroni’s multiple comparison test), and the expression level was either maintained or increased on day 3 (*P* < 0.05, Bonferroni’s multiple comparison test). The expressions of these genes did not increase on other substrates, regardless of ligand stimulation.

Compared to the weak immunofluorescent signals for TLR4 proteins in cells on a polystyrene culture plate and the SM titanium surface (Fig. 3C, arrows), the untreated cells on both titania nanosurfaces emitted strong and extensive signals for TLR4 proteins under a confocal laser microscope (Fig. 3C, double arrows). The intensity of TLR4-labeled signals on untreated cells was the highest on the NR2 titania surface, followed by the NR1 titania surface (Fig. 3C, box plots; *P* < 0.05, Games–Howell test).

The M1- or M2-induced cells, in addition to the untreated cells on the SM or MR titanium surfaces showed slight or no increase in the *TLR4* and *TLR2* expression levels, in serum-free medium on days 1 and 3 (Fig. 3D). In contrast, both titania nanosurfaces significantly upregulated the expression of these genes on days 1 and 3, even in serum-free medium (*P* < 0.05, Bonferroni’s multiple comparison test).

### Effects of titania nanosurfaces on phagocytic activity of macrophages

**Figure 4:**
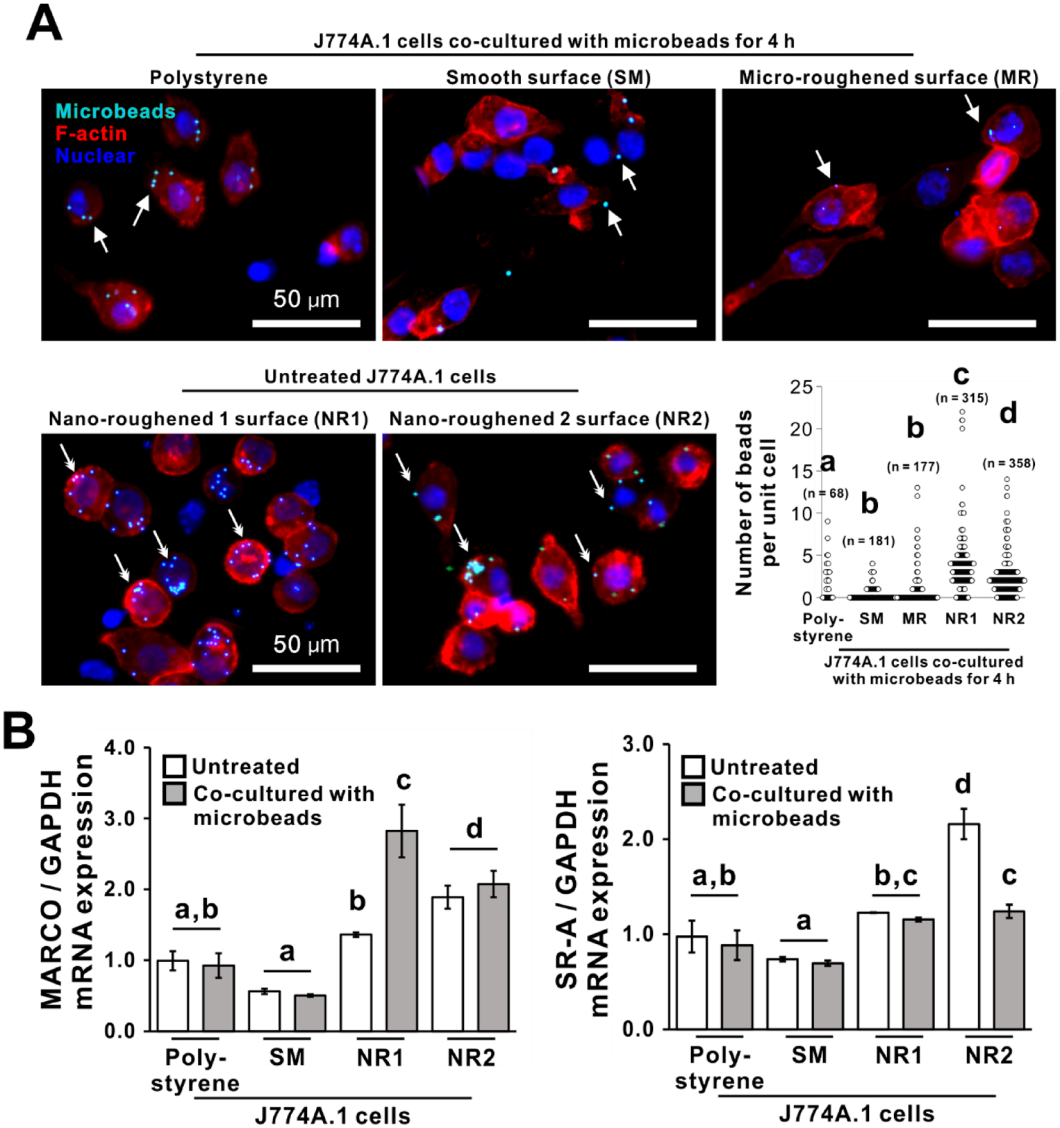
Effects of titania nanosurfaces on phagocytic activity of macrophage (**A**) Representative confocal laser microscopic images for F-actin (red), nuclear (blue) staining, and fluorescent microbeads (light blue); and dot plots for the number of beads per unit cell in day 1 J774A.1 cells co-incubated with microbeads for 4 h on a polystyrene culture plate or smooth (SM), micro-roughened (MR), nano-roughened (NR) 1 or 2 surfaces. (**B**) Gene expression of macrophage receptor with collagenous structure (*MARCO*) and class A macrophage scavenger receptors (*SR-A*) relative to glyceraldehyde 3-phosphate dehydrogenase (*GAPDH*) as analyzed by reverse transcription-polymerase chain reaction (RT-PCR) in the cells under different culture conditions. Data represented as mean ± standard deviation (SD; *N* = 68–358 in A, and *N* = 3 in B). Different letters indicate statistically significant differences (*P* < 0.05; Bonferroni multiple comparisons test). Note that (**A**) the microbead signals were stronger in macrophages on the NR1 or NR2 titania surfaces (double arrows), in contrast with weaker signals on other surfaces (arrows).

Under confocal laser microscope, J774A.1 cells co-incubated with fluorescent microbeads for 4 h demonstrated that microbeads overlapped on cell bodies more on both the titania nanosurfaces than on the polystyrene and SM titanium surfaces (Fig. 4A). The number of microbeads per unit cell was highest on the NR1 titania surface, followed by the NR2 titania surface (Fig. 4A, dot plots; *P* < 0.05, Bonferroni’s multiple comparison test). The expression of *MARCO* and *SR-4* for the recognition of biological and artificial foreign matter in J774A.1 cells was different upon ligand stimulation. M1 induction upregulated the expression of the *MARCO* but not that of *SR-A* (Fig. S1). The expression of these genes in the untreated cells was higher on both the titania nanosurfaces than on the SM titanium surface (Fig. 4B; *P* < 0.05, Bonferroni’s multiple comparison test). However, the expression did not increase further with the exposure to microbeads (*P* > 0.05, Bonferroni’s multiple comparison test), except for the cells on the NR1 titania surface (*P* < 0.05, Bonferroni’s multiple comparison test).

### No TLR stimulation required for macrophage activation by titania nanosurfaces

Zymosan, a TLR2 ligand, upregulated *TNF-α* and *IL-1β* expression in a concentration-dependent manner until 20 µg/mL in J774A.1 cells cultured on polystyrene at both 12 and 24 h of incubation (Fig. S2A; *P* < 0.05, Bonferroni’s multiple comparison test). Preincubation with a 10 μg/mL anti-TLR2 antibody for 1 h reduced *TNF-α, IL-1β*, and *TLR2* gene expression in J774A.1 cells cultured on polystyrene in fresh medium for 12 h (Fig. S2B; *P* < 0.05, Bonferroni’s multiple comparison test). The expression of these genes further reduced (*P* < 0.05, Tukey’s HSD test) or remained unchanged (*P* > 0.05, Tukey’s HSD test) even after co-incubation with 20 µg/mL zymosan for 12 h. However, *TLR4* expression was upregulated when preincubated with TLR2 antibody and further increased by subsequent co-incubation with zymosan (*P* < 0.05, Bonferroni’s multiple comparison test).

**Figure 5:**
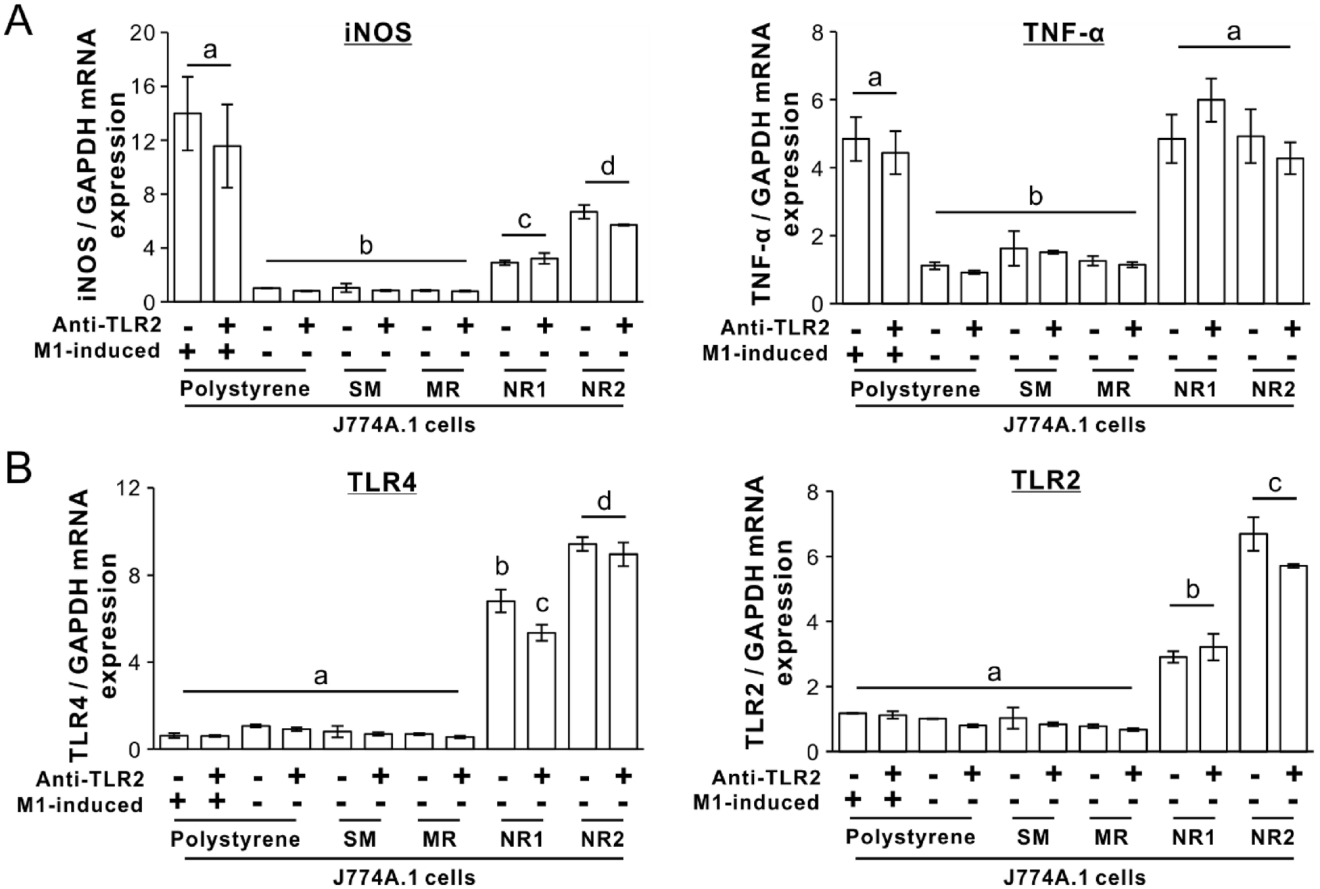
No TLR stimulation required for macrophage activation by titania nanosurfaces Gene expressions of (**A**) inducible nitric oxide synthase (*iNOS*), tumor necrosis factor-α (*TNF-α*), (**B**) toll-like receptor (*TLR*) *4*, and *TLR2* relative to glyceraldehyde 3-phosphate dehydrogenase (*GAPDH*) as analyzed by reverse transcription-polymerase chain reaction (RT-PCR) in J774A.1 cells preincubated with or without a 10 μg/mL anti-TLR2 antibody for 1 h and then cultured on a polystyrene culture plate or on smooth (SM), micro-roughened (MR), or nano-roughened (NR) 1 or 2 surfaces in fresh DMEM with or without M1-induction for 24 h. Data represented as means ± standard deviation (SD; *N* = 3). Different letters indicate statistically significant differences (P < 0.05; Tukey’s honestly significant difference [HSD] test in **A**, Bonferroni multiple comparisons test in **B**).

The expression of *iNOS* and *TNF-α* in the M1-induced cells on polystyrene or the untreated J774A.1 cells on both the titania nanosurfaces remained upregulated, even when the cells were preincubated with a 10 μg/mL anti-TLR2 antibody for 1 h (Fig. 5A; *P* > 0.05, Tukey’s HSD test). In contrast, the expression of these genes in M2-induced cells or in untreated cells on polystyrene or on SM or MR titanium surfaces was not upregulated, regardless of preincubation with anti-TLR2 antibody (*P* > 0.05, Tukey’s HSD test). However, *TLR2* and *TLR4* gene expression was upregulated in the cells on both the titania nanosurfaces, regardless of preincubation with the anti-TLR2 antibody (Fig. 5B; *P* < 0.05, Bonferroni’s multiple comparison test). Conversely, preincubation with anti-TLR2 antibody hardly affected the expression of these genes, regardless of substrate type or M1-induction (*P* < 0.05, Bonferroni’s multiple comparison test).

### Additional effects of ligand stimulation on macrophage activation by titania nanosurfaces

**Figure 6:**
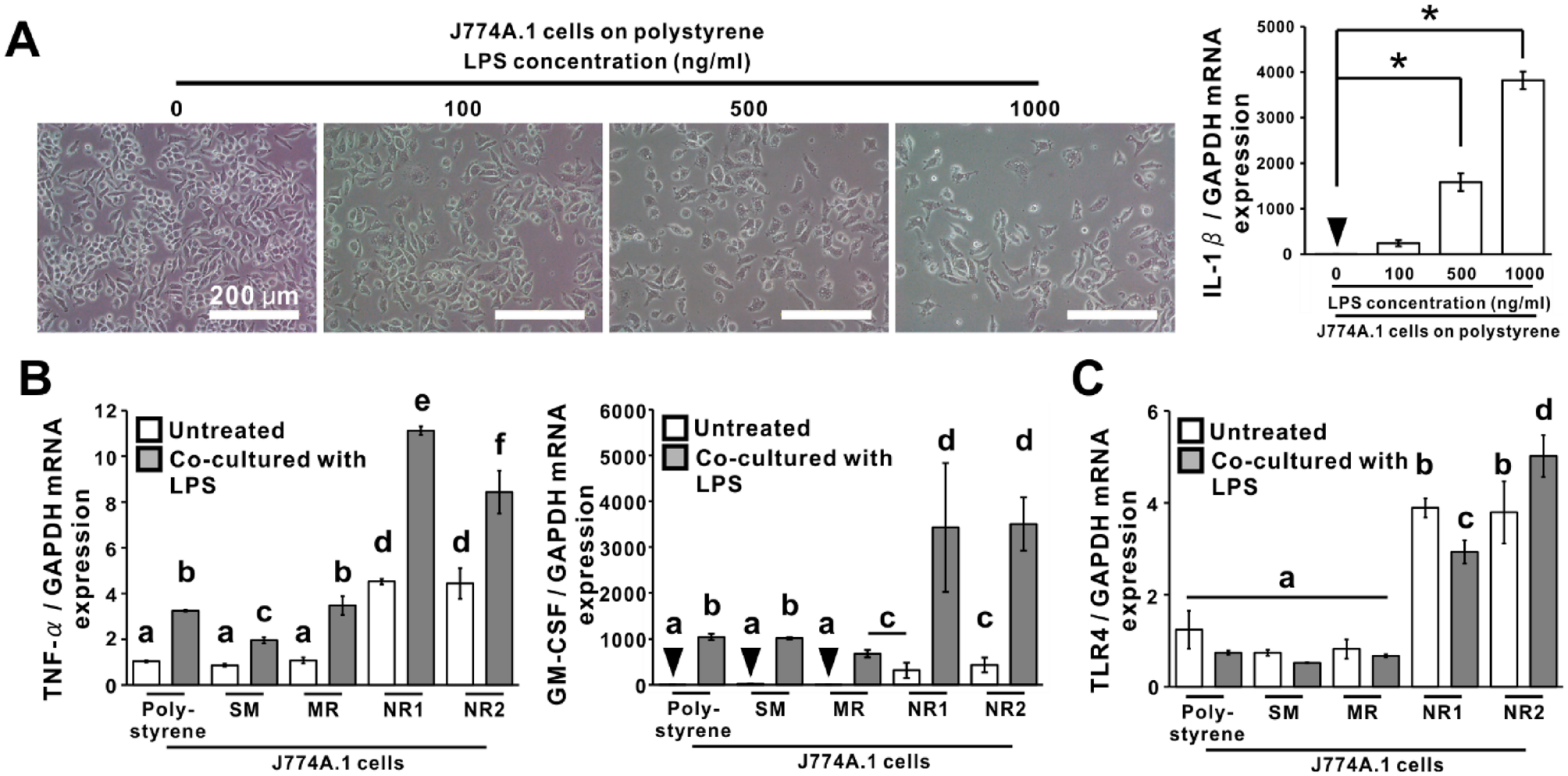
Additional effects of ligand stimulation on macrophage activation by titania nanosurfaces (**A**) Representative microscopic images of J774A.1 cells co-incubated with the various concentrations of lipopolysaccharide (LPS; 100, 500, and 1000 ng/mL) on a polystyrene culture plate for 24 h. A histogram shows the gene expression of interleukin-1 beta (*IL-1β*) relative to glyceraldehyde 3-phosphate dehydrogenase (*GAPDH*), as analyzed by reverse transcription-polymerase chain reaction (RT-PCR), in various concentrations of LPS. Gene expressions of (**B**) tumor necrosis factor-α (*TNF- α*), granulocyte–macrophage colony-stimulating factor (*GM-CSF*), and (**C**) toll-like receptor 4 (*TLR4*) genes expression relative to *GAPDH*, as analyzed by RT-PCR, in J774A.1 cells co-cultured with or without 100 ng/mL LPS on the polystyrene or smooth (SM), micro-roughened (MR), or nano-roughened (NR) 1 or 2 surfaces. Data represented as mean ± standard deviation (SD; *N* = 3). Different letters indicate statistically significant differences (*P* < 0.05; Dunnett’s test in **A** and Bonferroni multiple comparisons test in **B** and **C**).

To determine the LPS concentration for ligand stimulation, J774A.1 cells were co-incubated with various concentrations of LPS for 24 h on a polystyrene culture plate (Fig. 6A). The addition of LPS ≥ 500 ng/mL reduced the quantity of attached cells and increased the *IL-1β* expression (*P* < 0.05, Dunnett’s test) in the J774A.1 cultures in a concentration-dependent manner. Therefore, 100 ng/mL LPS was selected as the appropriate concentration for ligand stimulation of J774A.1 cells on titanium or titania surfaces. *TNF-α* and granulocyte–macrophage colony-stimulating factor (*GM-CSF)* expression in J774A.1 cells on, polystyrene culture plate and on SM and MR titanium surfaces were upregulated by LPS addition (Fig. 6B; *P* < 0.05, Bonferroni’s multiple comparison test). Both the titania nanosurfaces upregulated the expression of these genes without LPS addition (*P* < 0.05, Bonferroni’s multiple comparison test) to levels comparable to or higher than those in other cultures with LPS addition. Moreover, the upregulation of these genes by both titania nanosurfaces was further amplified significantly by LPS addition (*P* < 0.05, Bonferroni’s multiple comparison test). In contrast, LPS addition only slightly altered the upregulation of *TLR4* expression by titania nanosurfaces (Fig. 6C; *P* < 0.05, Bonferroni’s multiple comparison test) or unchanged the expression on the other substrates (*P* > 0.05, Bonferroni’s multiple comparison test).

### Effects of titania nanosurfaces on adhesion behavior of macrophages

**Figure 7:**
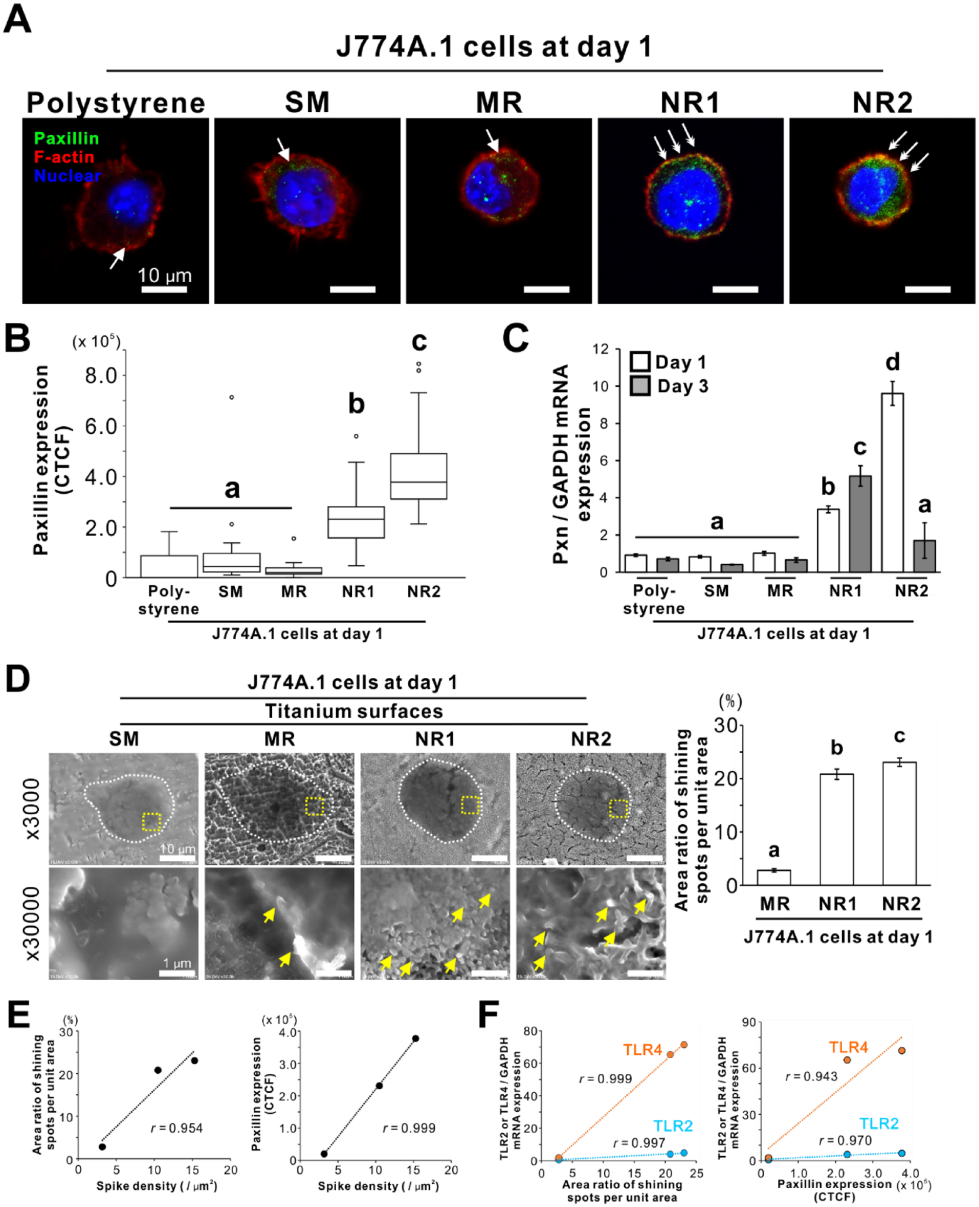
Effects of titania nanosurfaces on adhesion behavior of macrophages Representative confocal laser microscopic images of (**A**) F-actin (red), nuclear (blue), and paxillin (green) staining and (**B**) the intensity of paxillin-labeled signals in J774A.1 cells cultured on a polystyrene culture plate or smooth (SM), micro-roughened (MR), or nano-roughened (NR) 1 or 2 surfaces on day 1. (**C**) Gene expression of paxillin (*Pxn*) relative to glyceraldehyde 3-phosphate dehydrogenase (*GAPDH*), as analyzed by reverse transcription-polymerase chain reaction (RT-PCR), in the cells under the corresponding culture conditions on days 1 or 3. (**D**) Representative scanning electron microscopy (SEM) images and the area ratio of shining spots per unit area within the peripheral region of the cell body in the cells cultured on each surface on day 1. (**E**) Scatter plots indicating a positive linear relationship between the area ratio of shining spots per unit area or the intensity of paxillin-labeled signals and the spike density or **(F)** the gene expression levels of toll-like receptor (*TLR*) *4* and *TLR2* relative to *GAPDH*, as analyzed by RT-PCR, in macrophages on the MR, NR1, and NR2 surfaces. Data represented as box plots (*N* = 15–52 in **B**) or mean ± standard deviation (SD; *N* = 3 in **C** and *N* = 5–8 in **D**). Different letters indicate statistically significant differences (*P* < 0.05; Tukey’s honestly significant difference [HSD] test in **B**, Bonferroni multiple comparisons test in **C**, and Games–Howell test in **D**). CTCF, Corrected total cell fluorescence. Note (**A**) the strong paxillin signals in macrophages on the NR1 or NR2 titania surfaces (double arrows) in contrast with fewer signals on the other surfaces (arrows), and (**D**) the shining spots (yellow arrows) corresponding to the vertices of surface spikes digging into the peripheral parts of the macrophage cell body (yellow dashed rectangular within the white dashed circle) are increased on the NR1 and NR2 titania surfaces compared to that on the MR titanium surface.

A slight increase in the expression of a focal adhesion-associated adaptor protein, paxillin, was observed in the untreated J774A.1 cells on a polystyrene culture plate and the SM and MR titanium surfaces on day 1 (Fig. 7A, arrows). In contrast, paxillin was highly expressed in untreated cells on both the titania nanosurfaces (Fig. 7A, double arrows). The intensity of paxillin-labeled signals was higher in cells on both the titania nanosurfaces, particularly on the NR2 titani surface than on the other surfaces (Fig. 7B; *P* < 0.05, Tukey’s HSD test). The paxillin gene (*Pxn*) expression in the untreated J774A.1 cells was significantly upregulated on both the titania nanosurfaces at days 1 and 3 more than the levels in the cells on the other substrates (Fig. 7C; *P* < 0.05, Bonferroni’s multiple comparison test).

SEM observations of the day 1 culture showed that the MR, NR1, and NR2 surfaces made shining spots at the periphery of the cell surface by impaling the cell body with the vertices of spikes (Fig. 7D, arrows). In particular, both the titania nanosurfaces presented seven or more times the shining spots than the MR titanium surface (Fig. 7D, histogram; *P* < 0.05, Games–Howell test), with NR2 titania surface having a slightly higher shining value than the NR1 titania surface (*P* < 0.05, Games–Howell test). For all roughened surfaces, the mean value of the area ratio of shining spots per unit area (Fig. 7D) and the median value of paxillin expression (Fig. 7B) showed a positive linear relationship (Fig. 7E) with the mean values of the spike density (Fig. 1A). These values also showed a positive linear relationship (Fig. 7F) with the mean values of the expression levels of *TLR2* and *TLR4* in cells on each roughened surface (Fig. 3B).

### Involvements of nanotopography, but not other surface properties, in macrophage activation by titania nanosurfaces

**Figure 8:**
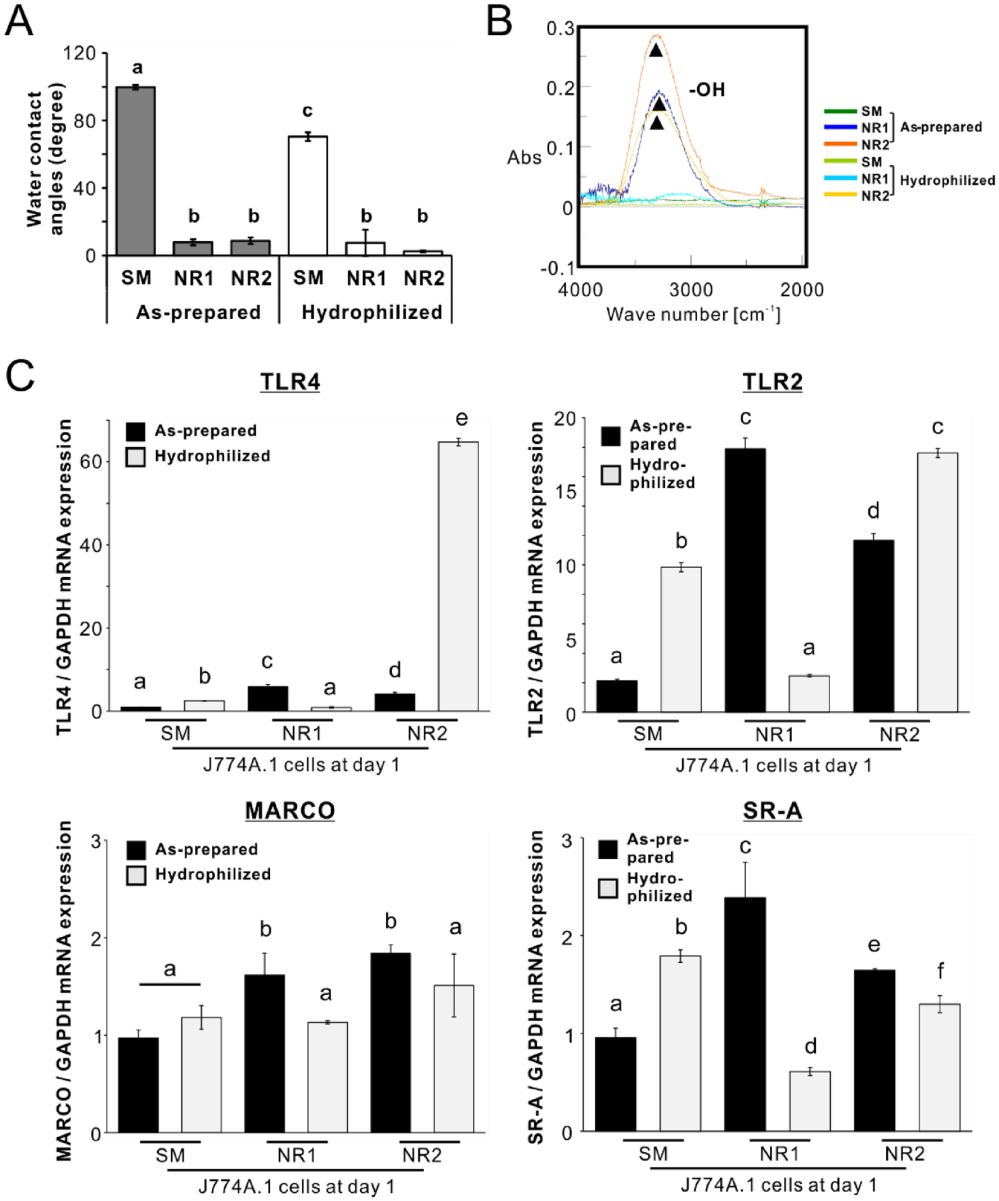
Involvement of nanotopography in macrophage activation by titania nanosurfaces **(A)** Water contact angles as determined by the sessile drop method. (**B**) Fourier transform infrared (FTIR) spectra of titanium sheets with smooth (SM) and nano-roughened (NR) 1 and 2 surfaces before (as-prepared) and after ultraviolet (UV) irradiation treatment. The black arrows in the FTIR spectra indicate spectral peaks corresponding to hydroxyl groups (–OH). (**C**) Gene expressions of toll-like receptor (*TLR*) *4*, *TLR2*, macrophage receptor with collagenous structure (*MARCO*), and class A macrophage scavenger receptors (*SR-A*) relative to glyceraldehyde 3-phosphate dehydrogenase (*GAPDH*), as analyzed by reverse transcription-polymerase chain reaction (RT-PCR), in J774A.1 cells cultured for 3 days on SM, NR1, and NR2 surfaces pre-irradiated and not irradiated with UV. Data represented as the mean ± standard deviation (SD; *N* = 3). Different letters indicate statistically significant differences (*P* < 0.05; Tukey’s honestly significant difference [HSD] test in **A** and Bonferroni’s multiple comparison test in **C**).

UV irradiation reduced the water contact angles on the SM titanium surface to 70° (hydrophilic status; Fig. 8A; *P* < 0.05, Tukey’s HSD test) and kept the angles less than 10° (superhydrophilic) on both titania nanosurfaces (*P* > 0.05, Tukey’s HSD test). FTIR analysis demonstrated that UV irradiation did not influence the status of the hydroxyl groups on the SM and NR2 titania surfaces but erased the hydroxyl groups on the NR1 titania surfaces (Fig. 8B).

In the as-prepared states, both titania nanosurfaces consistently upregulated *TLR4*, *TLR2 MARCO*, and *SR-A* expression levels in untreated J774A.1 cells on day 3 (Fig. 8C; *P* < 0.05, Bonferroni’s multiple comparison test). UV pre-irradiation on the titanium and titania surfaces upregulated the expression of these genes in macrophages (*P* < 0.05, Bonferroni’s multiple comparison test), except for the NR1 titania surfaces, which always reduced the expression of these genes post UV pre-irradiation (Fig. 8C; *P* < 0.05, Bonferroni’s multiple comparison test). In particular, *TLR4* expression on NR2 titania surfaces was markedly upregulated after UV pre-irradiation. UV-irradiated SM titanium surfaces upregulated the expression of these marker genes in macrophages to a level close to those on both types of as-prepared titania nanosurfaces (*P* < 0.05, Bonferroni’s multiple comparison test).

## Discussion

Both the NR1 and NR2 titania surfaces markedly upregulated the expression of M1 (*iNOS* and *TNF-α*), but not M2 markers (*Arg1*) (Figs. 2A,B and 6B), in the mouse macrophage cell line J774A.1, to levels close to those in M1-induced cells. A circular cell shape and strong iNOS expression were observed in these cells (Fig. 2C). Cytotoxicity was not observed on cells grown on the NR1 and NR2 surfaces (Fig. 2D). *TNF-α* expression in J774A.1 cells on polystyrene plate and on the SM or MR titanium surfaces was also upregulated by LPS addition, but the levels were lesser than that in macrophages on both the titania nanosurfaces, even without LPS addition (Fig. 6B). These observations were consistent with the results of our previous study, which showed that the same titania nanosurfaces polarized mouse-derived macrophage cell line into M1-like type, characterized by a circular cell shape with filopodium-like projections and production of osteoclastogenesis inhibitory factors^27^. These M1 markers not only regulate the immunological system but also activate macrophage phagocytosis. TNF-α might activate the phagocytic or chemotactic activity of macrophages through autocrine signaling^31^. Microbes engulfed by macrophages are intracellularly killed by reactive oxygen species via the iNOS reaction^32^. Both the titania nanosurfaces markedly stimulated the phagocytic activity of macrophages compared to the SM and MR titanium surfaces (Fig. 4A). The expression of phagocytosis-related receptors such as *TLR2, TLR4* (Figs. 3A-C, 6C, 7C)*, MARCO,* and *SR-A* (Fig. 4B and Fig. 7C) in macrophages was increased at both the gene and protein levels when cultured on both the titania nanosurfaces without any ligand stimulation. The concentrations of endotoxins detected on all surface types were extremely low, below the limit value to exert biological effects in cell culture experiments (0.03 EU/mL)^33^ (Fig. 2E). Both nanosurfaces upregulated *TLR4* and *TLR2* expression in untreated cells even in serum-free medium (Fig. 3D), where the possibility of deposition of unknown ligands onto the surfaces with nanotopography^34^ should have not occurred. Phagocytosis-related receptors induce the formation of phagocytic cups through actin remodeling and membrane protrusion extension. Phagosomes are formed after the phagocytic cup is sealed and gradually mature through a series of fusion and fission events from an early phagosome to the late phagosome^35^. TLR2 and TLR4 are known to be involved in phagocytosis in murine macrophages. In early phagosome formation, TLR2 and TLR4 are recruited into the phagocytic cup to activate signaling pathways mediated by toll/interleukin-1 receptor homology (TIR) domain-containing adaptor proteins for phagosome maturation^36–39^. Therefore, both the titania nanosurfaces activated phagocytic activity of macrophages through M1-like polarization directly with surface properties.

The expression of the phagocytosis-related genes, except *MARCO*, was not upregulated by ligand stimulation in most cases, including M1- or M2-inducing reagents (Fig. 3A, B, D, and Fig. S1), TLR2-ligand zymosan (Fig. S2), and TLR4-ligand LPS (Fig. 6C), while *MARCO* expression was by M1-induction (Fig. S1). Microbeads did not upregulate *MARCO* and *SR-A* expression, regardless of substrate type (Fig. 4B). In addition, upregulation of *iNOS*, *TNF-α*, *TLR2*, and *TLR4* expression was not blocked in J774A.1 cells on both the titania nanosurfaces by preincubation with anti-TLR2 antibodies (Fig. 5). These observations indicated that the upregulation of phagocytosis-related gene expression in macrophages on titania nanosurfaces was independent of the ligand stimulation and was not due to the recognition of the titania surface itself as a foreign matter. Therefore, titania nanosurfaces activated macrophage phagocytosis as a result of feedback after non-inflammatory stimulation with surface properties.

**Figure 9:**
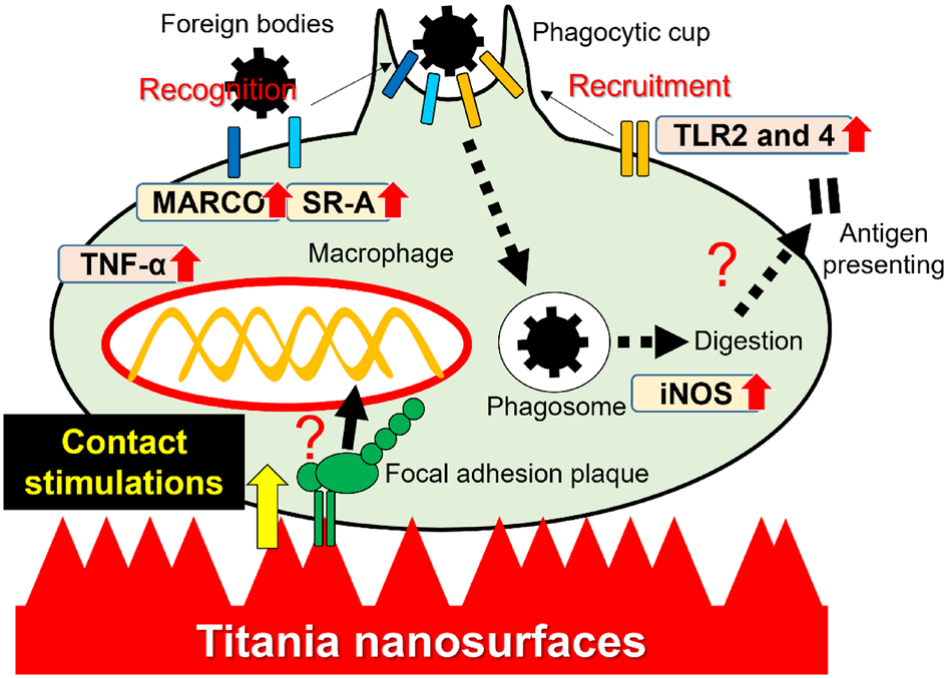
Activation of macrophage phagocytosis through contact stimulation by nanospikes on titania nanosurfaces Scheme showing a possible mechanism underlying the activation of macrophage phagocytosis by titania nanosurfaces. Titania nanosurfaces polarize macrophages into an M1-like type via topographic cues ^27^ to promote the production of pro-inflammatory mediators such as iNOS and TNF-α. Meanwhile, the nanospikes on titania nanosurfaces promote the formation of focal adhesion plaques and provide physical stimuli to the macrophage cell body. These contact stimulations by titania nanospikes might trigger the expression of phagocytosis-related receptors, such as TLR2, TLR4, MARCO, and SR-A, and eventually activate phagocytosis and the subsequent processes. Pro-inflammatory mediators may help in macrophage chemotaxis or intracellular digestion of foreign bodies. iNOS, inducible nitric oxide synthase; TNF-α, tumor necrosis factor-alpha; TLR, toll-like receptor; MARCO, macrophage receptor with collagenous structure; SR-A, class A macrophage scavenger receptor.

Focal adhesion plaques are crucial for sensing the physical cues of surface topography, even for macrophages^40^. Focal adhesion-related signaling pathways can regulate pro-inflammatory reactions in macrophages by interacting with an adaptor protein that transduces TLR2 or TLR4 signals^41–43^. In macrophages, the focal adhesion molecules, integrins, regulate TLR2-mediated recognition of microbial components^44^. A certain type of integrin regulates the TLR4-mediated pro-inflammatory response in macrophages by regulating the expression of CD14^45^ which initiates TLR4-mediated endocytosis^46^. Expression of the focal adhesion adaptor protein, paxillin, in J774A.1 cells increased on both the titania nanosurfaces, particularly on the NR2 titania surfaces at gene and protein levels on day 1 (Fig. 7A-C). Titania nanosurfaces can enhance cellular attachment^47^ by assembling focal adhesion plaques on the vertices of the surface^48^. In this study, both types of titania nanosurfaces were markedly higher in spike density than the MR surfaces; NR2 titania surfaces had 1.3 and 4 times more spike density than the NR1 and MR surfaces, respectively (Fig. 1A). Except for the micron-scale irregularity of the MR titanium surfaces, the NR2 titania surfaces had the highest vertical roughness values at the submicron-scale which were two times or more than the NR1 or SM titanium surfaces (Fig. 1B). Paxillin expression showed a positive linear relationship with spike density (Fig. 7E), as well as with the *TLR4* and *TLR2* expression levels (Fig. 7F) for the roughened surfaces used in this study. The area ratio of shining spots per unit area also showed a positive linear relationship with spike density (Fig. 7E) and with the *TLR4* and *TLR2* expression levels (Fig. 7F). In addition, the nanospikes on the NR titania surfaces stuck out the cell body of macrophages, as shown by the shining spots on the cell surface, consistent with the vertices under SEM (Fig. 7D and E). 2D NR titania surfaces, such as nanotube arrays of titania^49,50^, nanochannels of titanium-zirconia alloys^51^ and pure titanium coatings^52^ regulated macrophages or cell lines to reduce pro-inflammatory cytokines and chemokines production. The vertices of these nanosurfaces had relatively round ends, in contrast with the spikey vertices on the NR titania surfaces used in this study or the 1D vertical nanostructures for the activation of lymphocytes^20–22^. Contact stimulation by nanospikes on the titania nanosurfaces might have induced the expression of phagocytosis-related receptors and eventually the phagocytic activity of macrophages through direct physical stimuli or promoted the formation of focal adhesion plaques or both (Fig. 9).

Both the titania nanosurfaces were superhydrophilic with the hydroxyl group, in contrast to the SM surface which was hydrophobic without the functional group (Fig. 1C and D). After UV irradiation-mediated hydrophilization, the NR1 and NR2 titania surfaces maintained superhydrophilicity, but NR1 lost the hydroxyl group, unlike NR2 which retained the functional group (Fig. 8A and B). The SM titanium surface turned hydrophilic even without hydroxyl groups, following UV treatment (Fig. 8A and B). UV treatment further upregulated the expression of *TLR2* and *TLR4* on the NR2 titania surfaces, whereas it downregulated phagocytosis-related gene expression on the NR1 titania surfaces (Fig. 8C). The expression of these genes was upregulated even on the UV-irradiated SM surface, although not as much as on the as-prepared titania nanosurfaces (Fig. 8C). The hydrophilicity and hydroxyl groups potentially facilitate cell attachment^53,54^. However, UV irradiation-mediated hydrophilization did not alter the expression of M1 gene markers in macrophages on the titania nanosurfaces^27^. These observations suggest that UV irradiation might have induced secondary changes in surface properties, influencing macrophage phagocytic gene expression.

Due to the photocatalytic effect^55^, UV irradiation produces oxygen vacancies on titanium or titania surfaces by decomposing the oxygen-containing hydrocarbon molecules that are naturally deposited on these surfaces^56^. Oxygen vacancies generally form hydroxyl groups on titanium or titania surfaces via the adsorption of water molecules from the atmosphere, leading to a highly hydrophilic surface^57^. However, the UV treatment used in this study maintained the hydrophilicity of the SM or NR1 surfaces without the presence of hydroxyl groups (Fig. 8A and B). The UV-mediated photocatalytic effect induced a positive charge on the titanium or titania surfaces by generating electron–hole pairs via the transition of electrons from the valence band to the conduction band^58^. Electrically polarized material surfaces exert hydrophilicity^59^. In this study, surface analyses or culture experiments on UV-irradiated surfaces were performed immediately after the treatment. UV-mediated electric excitation might have increased or maintained the hydrophilicity of titanium or titania surfaces^60^.

Furthermore, titanium or titania materials electrically excited by UV irradiation promotes cell adhesion to the surfaces^61^. However, cell adhesion decreases when the UV irradiation energy is exceeded^62^. Spiky vertices of titanium or titania surfaces allow the concentration of the inherent surface charge on the vertices^63^ to not only adsorb adhesion proteins but also physically stimulate various types of cell bodies^5,24^. Electric stimulation causes actin polarization and clustering of phagocytic receptors to stimulate the phagocytic activity in macrophages^64^. Synergistic effects of titania nanospikes and physical surface properties, particularly electric potentials, on macrophage behavior could be of great interest for future research.

This study demonstrated a novel finding that titania nanosurfaces with nanospikes activate macrophage phagocytic activity, independent of ligand stimulation. This titania nanosurface-mediated macrophage activation might be augmented by ligand stimulation, as shown by further upregulation of *TNF-α* and *GM-CSF* expression in cells on titania nanosurfaces when co-incubated with LPS (Fig. 6B). Titania nanosurfaces may be applicable as a 2D patterned platform to create an artificial culture environment that mimics an immune disease caused by excessive macrophage phagocytosis, such as Alzheimer’s disease^65^. In addition, macrophages on titania nanosurfaces inhibit the differentiation of preosteoclasts via production of an inhibitory factor for osteoclastogenesis^27^ such as *GM-CSF*^66^. Titania nanosurface implants may prevent inflammatory osteolysis through activation of phagocytosis and increased production of an inhibitory factor for osteoclastogenesis in macrophages, even after an infection. However, the influence of macrophage cell shape on polarization might differ among cell types. As shown in primary bone marrow-derived^16^, peritoneal macrophages^67^, and the J774A.1 cell line^27^, mouse-derived macrophages tend to be polarized to the M1 or M2 type when they are circular or spindle-shaped, respectively. In contrast, the association between cell shape and the type of polarization is different in human-derived macrophages. Inconsistent cell shapes, such as fried egg-like^68^ or elongated spindle shapes^69^ were observed in M1-polarized macrophages that differentiated from human peripheral blood monocytes in vitro. The human monocytic leukemia cell line tends to have a spindle-like cell shape upon M1 induction^70^. Therefore, it is necessary to verify the effects of titania nanosurfaces on human-derived macrophages in the future^71^. However, this novel approach to tune immune cell functions with contact stimulation by a 2D patterned platforms would provide important information toward the advancement of therapeutic biomaterials or diagnostic tools for immunological intervention.

## Conclusion

The 2D titania nanosurfaces created by alkali etching treatment activate macrophage phagocytosis through the upregulation of phagocytosis-related receptors by nanospike-mediated ligand-independent contact stimulation assisted by surface physical properties.

## Supplementary Information

Supplementary Information.

## Data Availability

The raw and processed datasets generated and/or analysed during the current study are available in the Mendeley Data repository, https://doi.org/10.17632/bvpvk39svk.1.
