# Supplementary material for: Titania nanospikes activate macrophage phagocytosis by ligand-independent contact stimulation
Source: Sci Rep. 2022 Jul 18;12:12250. doi: 10.1038/s41598-022-16214-2 (PMC9293906; doi:10.1038/s41598-022-16214-2)
Supplement: Supplementary file 1 — Supplementary Information. [file 41598_2022_16214_MOESM1_ESM.docx]

*Sci Rep.* [Original Research]

**Titania nanospikes activate macrophage phagocytosis by ligand-independent contact stimulation**

Nadia Kartikasari^1^, Masahiro Yamada^1^*, Jun Watanabe^1^, Watcharaphol Tiskratok^1^, Xindie He^1^ and Hiroshi Egusa^1, 2^

^1^ *Division of Molecular and Regenerative Prosthodontics, Tohoku University Graduate School of Dentistry, Sendai, Miyagi, Japan.*

^2^ *Center for Advanced Stem Cell and Regenerative Research, Tohoku University Graduate School of Dentistry, Sendai, Miyagi, Japan.*

**Keywords:** macrophage polarization, nanotopography, proinflammatory cytokines, osteoimmunology, toll-like receptors, surface modification

Those authors have no conflict of interest.

*** Address corresponding to:**

Masahiro Yamada, DDS, PhD

Division of Molecular and Regenerative Prosthodontics,

Tohoku University Graduate School of Dentistry

4-1 Seiryo-machi, Aoba-ku, Sendai, 980-8575, Japan

tel: +81-22-717-8363; fax: +81-22-717-8367

E-mail: [masahiro.yamada.a2@tohoku.ac.jp](mailto:masahiro.yamada.a2@tohoku.ac.jp)

Hiroshi Egusa, DDS, PhD

Division of Molecular and Regenerative Prosthodontics,

Tohoku University Graduate School of Dentistry

4-1 Seiryo-machi, Aoba-ku, Sendai, 980-8575, Japan

tel: +81-22-717-8363; fax: +81-22-717-8367

E-mail: [egu@tohoku.ac.jp](mailto:egu@tohoku.ac.jp)


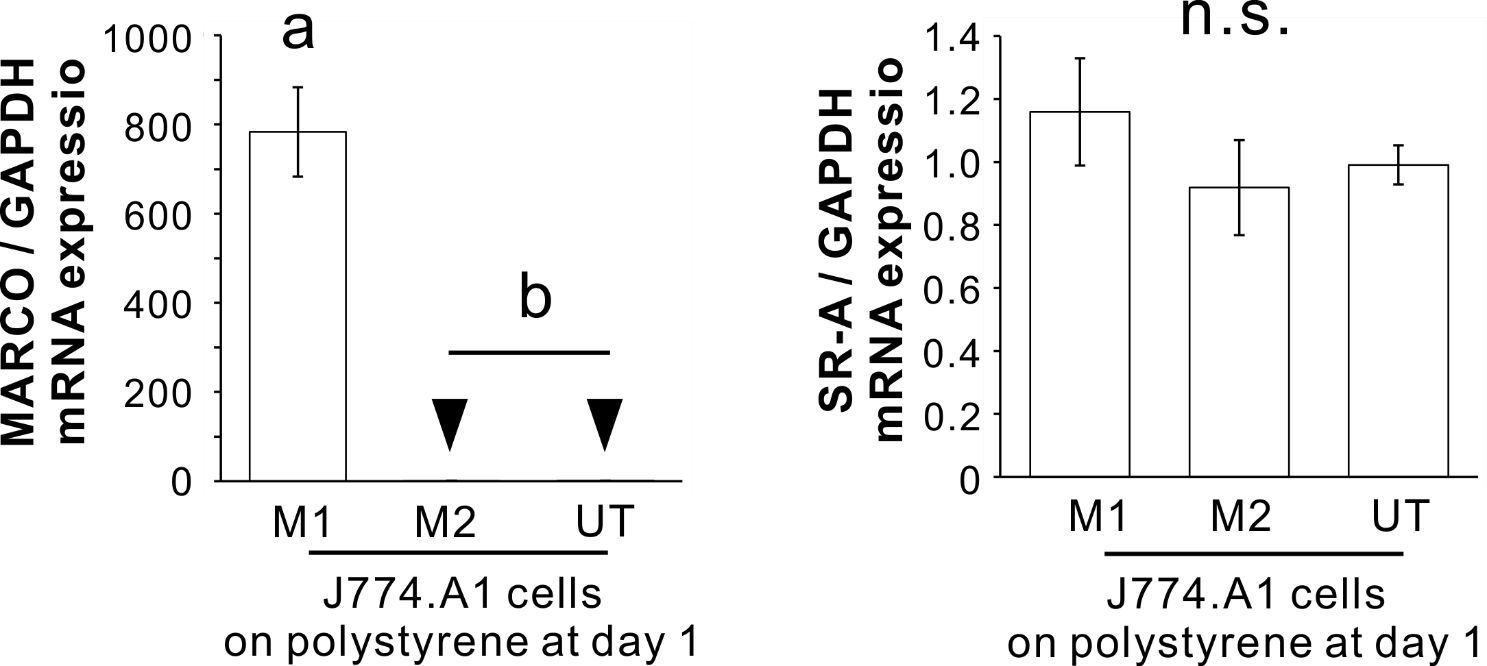


**Fig. S1. Effects of polarization on expression of phagocytosis-related receptors in macrophages**

Gene expression of macrophage receptor with collagenous structure (*MARCO*) and class A macrophage scavenger receptors (*SR-A*) relative to glyceraldehyde 3-phosphate dehydrogenase (*GAPDH*), as analyzed by reverse transcription-polymerase chain reaction (RT-PCR), in J774A.1 cells cultured on polystyrene with or without M1 or M2-induction for 24 h. Data represented as the mean ± standard deviation (SD; *N* = 3). Different letters indicate statistically significant differences (*P* < 0.05; Tukey’s honestly significant difference [HSD] test). UT, untreated cells; M1, M1-induced cells; M2-induced cells; n.s., non-significant difference.


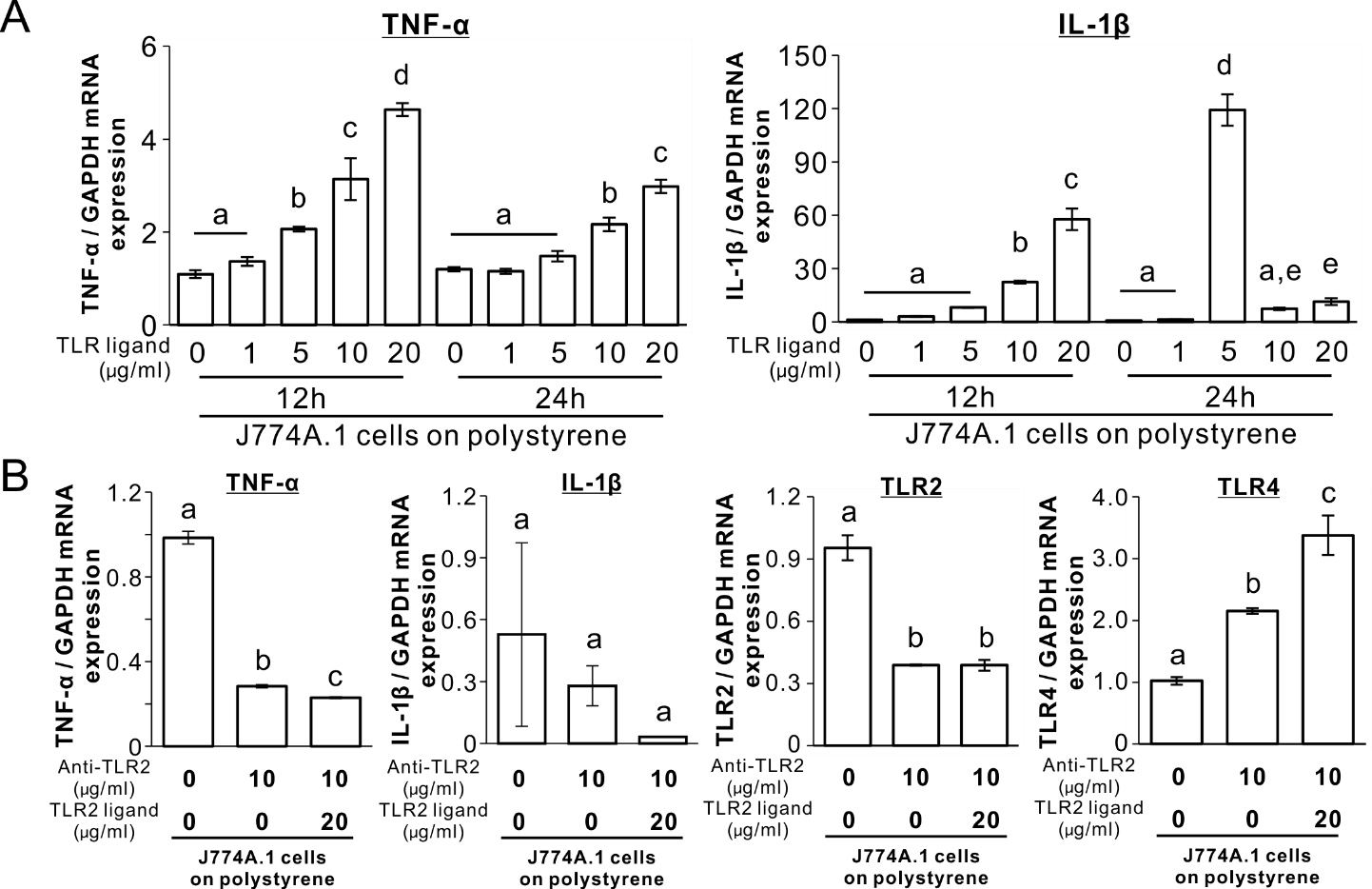


**Fig. S2.** **Determination of anti-TLR2 antibody concentration using zymosan**

(**A**) Gene expressions of tumor necrosis factor-α (*TNF-α*) and interleukin-1 beta (*IL-1β*) relative to glyceraldehyde 3-phosphate dehydrogenase (*GAPDH*), as analyzed by reverse transcription-polymerase chain reaction (RT-PCR), in J774A.1 cells cultured on polystyrene without or with the addition of 1, 5, 10, and 20 µg/mL toll-like receptor (TLR) 2 ligand, zymosan, for 12 and 24 h. (**B**) Gene expressions of *TNF-α*, *IL-1β*, *TLR2*, and *TLR4* relative to *GAPDH*, as analyzed by RT-PCR, in J774A.1 cells co-incubated with or without 10 µg/mL anti-TLR2 antibody on polystyrene for 1 h and then further cultured in a fresh medium without or with 20 µg/mL zymosan for 12 h. Data represented as mean ± standard deviation (SD; *N* = 3). Different letters indicate statistically significant differences (*P* < 0.05; Bonferroni multiple comparison test).

 **Table S1. A primer list for the SYBR-green-based PCR reactions**
